# Distorted Immunodominance by Linker Sequences or other Epitopes from a Second Protein Antigen During Antigen-Processing

**DOI:** 10.1038/srep46418

**Published:** 2017-04-19

**Authors:** AeRyon Kim, Tatiana N. Boronina, Robert N. Cole, Erika Darrah, Scheherazade Sadegh-Nasseri

**Affiliations:** 1 Department of Pathology, Johns Hopkins University School of Medicine, Baltimore, MD 21205, USA; 2 Mass Spectrometry and Proteomics Facility, Department of Biological Chemistry, The Johns Hopkins University School of Medicine, Baltimore, MD 21205, USA; 3 Division of Rheumatology, Department of Medicine, Johns Hopkins University School of Medicine, Baltimore, MD 21224, USA

## Abstract

The immune system focuses on and responds to very few representative immunodominant epitopes from pathogenic insults. However, due to the complexity of the antigen processing, understanding the parameters that lead to immunodominance has proved difficult. In an attempt to uncover the determinants of immunodominance among several dominant epitopes, we utilized a cell free antigen processing system and allowed the system to identify the hierarchies among potential determinants. We then tested the results *in vivo*; in mice and in human. We report here, that immunodominance of known sequences in a given protein can change if two or more proteins are being processed and presented simultaneously. Surprisingly, we find that new spacer/tag sequences commonly added to proteins for purification purposes can distort the capture of the physiological immunodominant epitopes. We warn against adding tags and spacers to candidate vaccines, or recommend cleaving it off before using for vaccination.

It is well known that the immune system focuses on and responds to very few representative immunodominant epitopes from pathogenic insults such as infectious agents and antigenic targets in autoimmune diseases, allergy, and cancer. Over the last decades, many studies have aimed to unravel the mechanisms of immunodominance. Among the hypotheses considered are intrinsic structural features of protein antigens^1^,^2^,^3^,^4^,^5^, sensitivity to proteases^6^,^7^,^8^,^9^,^10^,^11^, epitope affinity for MHC class II^12^, T cell precursor frequency^13^, T cell receptor affinity for peptide/MHC II^14^,^15^,^16^,^17^, contributions from HLA-DM or H-2M in mice (DM, from now on)^18^,^19^,^20^,^21^, and contributions from HLA-DO or H-2O in mice (DO, from now on)^22^,^23^,^24^. However, due to the complexity of the antigen processing pathway, pointing to a single mechanism as the driving force behind immunodominance has proved difficult.

For MHC class II presentation, antigens internalized from exogenous sources move through a series of endosomal compartments in APCs containing a suitable denaturing environment, the accessory molecules DM and DO, and a set of proteolytic and denaturing enzymes that generate and trim antigenic determinants^5^,^25^,^26^. Meanwhile, newly synthesized MHC class II molecules associate with the Class II invariant chain (Ii) in the ER, which guides the nascent MHC II through Golgi, trans Golgi, and to the compartments specialized for antigen processing and epitope selection, MHC II compartment (MIIC)^27^,^28^, where it meets the exogenous antigens. Ii is sequentially cleaved until only a short peptide, CLIP, remains that occupies the MHC II groove^29^. CLIP has been described as a surrogate peptide for completion of MHC class II folding^30^,^31^. Efficient displacement of CLIP from the MHC groove requires the accessory molecule DM^32^.

We developed a reductionist system for MHC class II molecules that utilizes only five purified protein components of the class II antigen presentation pathway, HLA-DR1 (DR1), DM, and three cathepsins, CatB, CatH and CatS that process the full-length protein antigens. Despite its minimalist nature, the system can capture physiologically relevant DR1-restricted immunodominant epitopes from several protein antigens, which proved immunodominant *in vivo*, as determined in DR1 transgenic mice and in DR1 + humans^21^,^33^,^34^. Moreover, the system was shown to be well suited for dissecting steps in antigen processing and in evaluating the factors that contribute to immunodominance^33^,^35^,^36^,^37^,^38^. By using this reductionist antigen processing system, we have learned some unexpected principles of immunodominance. We demonstrated that the immunodominant epitopes from several pathogens were highly sensitive to proteolysis unless protected by the groove of MHC II. We showed that for successful processing and presentation of the immunodominant epitopes, antigenic determinants must first be captured by MHC II, and then proteolysed by endo- and exoproteases^33^,^35^. These observations emphasize importance of structural factors to the accessibility, or constraints of epitopes captured by the groove of MHC II molecules^4^,^39^.

Contribution of structural factors to the immunodominance reminds us of the concept of ‘*epitope hierarchy’* described by Sercarz and colleagues, who suggested that while many epitopes within a protein antigen can bind to the groove of MHC II, some gain dominance over the others. Several molecular mechanisms were offered to explain this phenomenon^40^,^41^. Accordingly, one might envision that determinant hierarchy could occur during the processing of a single protein antigen or among few antigens all competing for binding to the same MHC II molecular groove.

Here, we have used our cell free antigen processing system to examine epitope hierarchy under two different conditions; a) among two different protein antigens whose immunodominant epitopes are known, and b) in single recombinant proteins that included spacer/affinity tag sequences for ease of purification as commonly practiced. We observed that when two proteins are used as immunogens *in vivo*, one dominant epitope prevailed as dominant even when it was at a lower quantity than the protein with the inferior epitope. More striking were data showing that by adding short spacers to a recombinant protein, the dominance of its natural epitopes was lost, being replaced by epitopes that partially include part or all of the spacer sequences. These observations have significant impacts for the design of recombinant proteins used in vaccines.

## Results

### An immune hierarchy is established when two different epitopes compete for the same HLA-DR1 molecules

**Figure 1:**
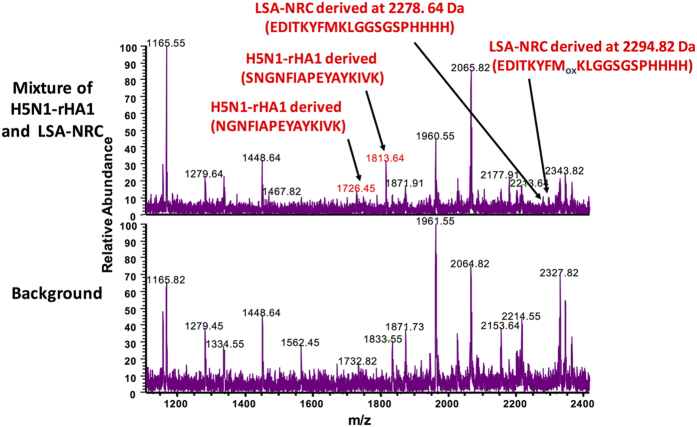
The cell-free system identifies dominant epitopes from both H5N1-rHA1 and LSA-NRC proteins when subjected to processing as a mixture. The mass spectra of peptides eluted from DR1. (**A**) Top spectrum shows peptides eluted from DR1 containing the mixture of H5N1-rHA1, LSA-NRC proteins, DR1, DM, CatB, CatH, and Cat S. (**B**) Bottom spectrum shows control background peaks in the absence of either protein antigens. See Methods section for details. Representative of three independent experiments/panel.

*The immune hierarchy* was originally conceptualized for the dominance of one epitope of an antigen over another, termed subdominant or cryptic^42^. We decided to examine this concept using our minimalist antigen processing system for two different proteins whose dominant epitopes were previously established. Previously, we have used this system for processing of single proteins from several sources, and have been able to obtain immunodominant epitopes that were proved physiological (Table 1) and refs 21 and 33. Now, we used a mixture of HA1 protein from influenza H5N1 strain, and a recombinant malaria protein Liver Stage Antigen 1 (LSA-NRC)^43^ at a ratio of 1:1, probing whether the cell-free system can detect immunodominant epitopes from both proteins. As previously shown, exogenous antigens bind MHC II groove as full proteins or large fragments. Hence, samples were assembled by mixing the two proteins with soluble DR1 and soluble DM for three hours before adding the cathepsins, i.e., CatB, CatH, and Cat S (Fig. 1). Three parallel samples served as controls: one included all the components of the reaction except for the protein antigens, and two other samples, included one protein antigen each. Samples were processed and the eluted peptides were subjected to MALDI mass spectrometry. When either H5N1-rHA1 or LSA-NRC were processed individually, the mass spec peaks that corresponded to their immunodominant epitopes (HA(260–274), and LSA(436–449) clearly stood out from the background peaks at m/z 1726, 1813, and 2202 Da for HA1 sample (Supplementary Fig. 1), and as previously shown^21^,^33^. Both HA1 derived peaks shared the same core dominant epitope, (NGNFIAPEYAYKIVK). A second control sample with recombinant LSA alone under identical conditions, led to the identification of LSA(436–449) at m/z 2278.6 and 2294.8 Da, which had been proven to be immunodominant as published previously^21^. However, in the sample with both proteins simultaneously processed by the cell free system, mass spectrometry results indicated only trace signal for LSA(436–449) containing peaks at 2278.6 Da and 2294.8 Da, but prominent H5N1-rHA1-derived peaks containing HA(259–274) at m/z 1726.5 and 1813.6 Da, which were distinguishable over the background peaks. Despite the clear differences in the height of the peaks from the two proteins, because MALDI is not a quantitative mass spectrometric method, we cannot claim that LSA(436–449) processing and capture was inferior to H5N1-rHA1 processing with this set of data.

**Figure 2:**
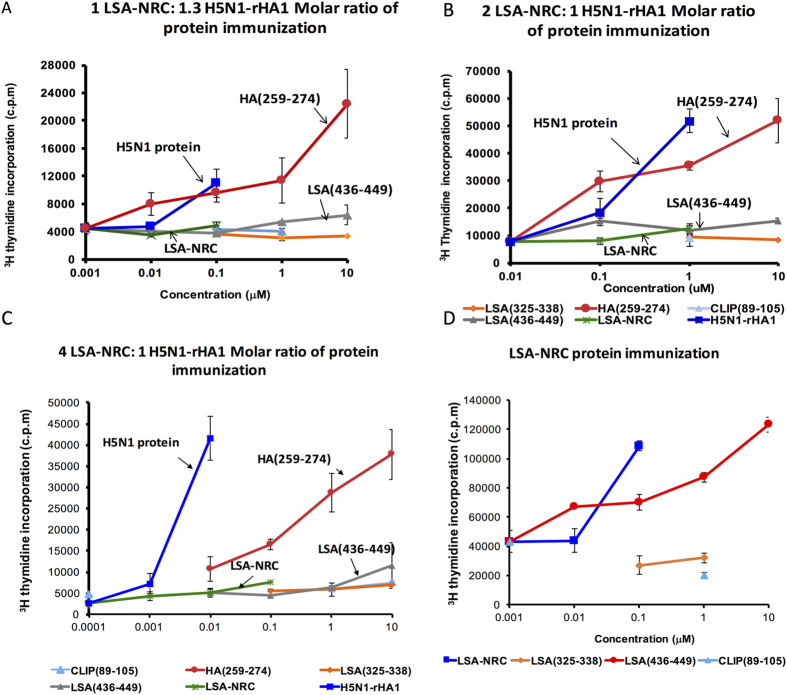
Immune hierarchy is established when two different dominant epitopes compete for the same HLA-DR1 molecules. (**A**–**C)** Proliferation of T cells isolated from DR1-transgenic mice immunized with both H5N1-rHA1 and LSA-NRC simultaneously in CFA at the base of tail. Cells were stimulated with H5N1-rHA1, LSA-NRC, CLIP(89–105), HA(259–274), LSA(436–449), and LSA(325–338) peptides *in vitro*. (**A**) DR1 mice were immunized with a 1 to 1.3 molar ratio of LSA-NRC to H5N1-rHA1. (**B**) Mice were immunized with a 2 to 1 molar ratio of LSA-NRC to H5N1-rHA1. (**C**) Mice were immunized with a 4 to 1 molar ratio of LSA-NRC to H5N1-rHA1. (**D**) Proliferation of T cells isolated from DR1-transgenic mice immunized with LSA-NRC in CFA at the base of tail. Cells were stimulated with LSA-NRC, CLIP(89–105), LSA(436–449), and LSA(325–338) peptides *in vitro*. Cellular proliferation was measure by [^3^H] thymidine incorporation. Error bars are defined as s.d. Representative of two-three independent experiments/panel.

To evaluate the hierarchy hypothesis and validate the cell free system depiction of the epitopes presented *in vivo,* HLA-DR1 transgenic mice were immunized with a mixture of HA1 and LSA-NRC at a ratio of 1.3:1.0. A recall T cell proliferation assay showed that T cells responded to the HA1 and its dominant epitope HA(259–274) only; no recall response was observed to LSA-NRC and LSA(436–449) (Fig. 2A). Those observations did not change even when the molar ratio of LSA-NRC to HA1 was increased to 2:1 (Fig. 2B) or even 4:1 (Fig. 2C). At the highest ratio of LSA-NRC:HA1 protein immunization, a slight response to the LSA epitope was detected. Because LSA-NRC protein is immunogenic when injected alone (Fig. 2D), the observed absence of T cell responses to LSA-NRC epitope^21^ cannot be explained by a ‘hole in CD4 T cell repertoire’ hypothesis^44^.

**Figure 3:**
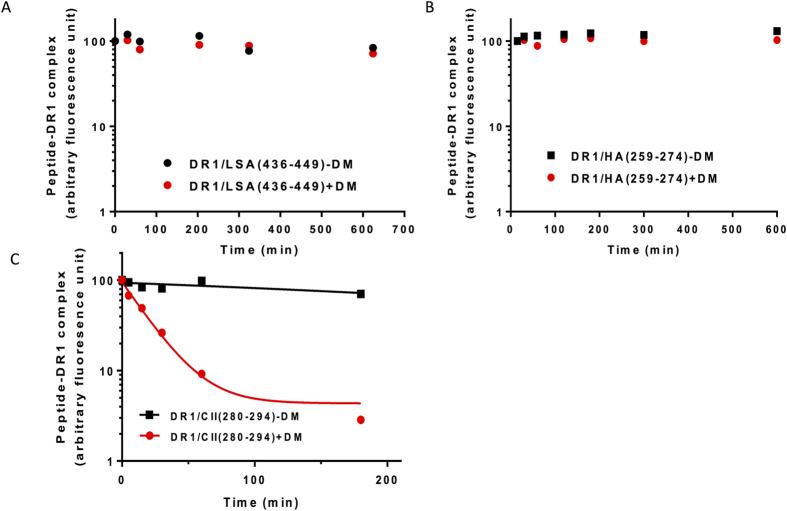
LSA-NRC and H5N1-rHA1 immunodominant epitopes are resistant to DM-mediated dissociation. (**A–C**) Dissociation assays of fluorescently labeled H5N1-HA(259–274) (**A**) LSA(436–449) (**B**), and collagen II(280–294) (**C**), from DR1 in the presence of 100 μM of unlabeled HA(306–318) at 37 °C for the indicated times in the absence (square), or presence of DM (circle). The fluorescence of the labeled complex before dissociation is arbitrarily assigned a value of 100, and fluorescence after dissociation is expressed as a fraction of fluorescence before dissociation. See Methods section for details. Representative of three-five independent experiments/panel.

We also tested the possibility of differences in the stability and/or DM resistance of HA(259–274)/DR1 versus LSA(436–449)/DR1 complexes. Dissociation kinetic assays with purified DR1 in complex with either HA(259–274) or LSA(436–449) peptides with or without DM were conducted per established protocols^45^. In brief, preformed complexes of fluorescent tagged HA(259–274) and LSA(436–449) with DR1 were allowed to dissociate in the presence of 100 μM unlabeled high affinity HA(306–318) peptide for the time shown on the x-axis and the unbound peptides were removed by G-50 size separation spin columns. The fluorescent signal associated with the bound eluted complexes were measured. The results shown in Fig. 3A,B demonstrate that both peptides form stable complexes with DR1 and are DM-insensitive. A control dissociation reaction for collagen II dominant epitope in complex with DR1 (Fig. 3C) with or without DM showed rapid dissociation in the presence of DM, hence validating that lack of dissociation of dominant epitopes from HA1 and LSA proteins was not due to lack of DM function or poor choice of an assay.

**Figure 4:**
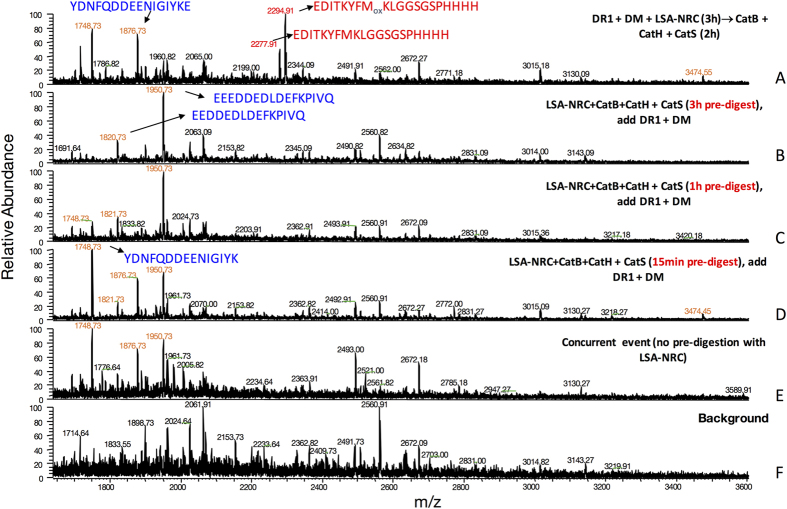
LSA-NRC derived immunodominant epitope is sensitive to cathepsin digestion. (**A–F**) Mass spectra of peptides eluted from DR1 are shown. (**A**) LSA-NRC protein was incubated with DR1 and DM for 3 hours first, and then CatB, CatH, and Cat S were added for additional 2 hours of incubation. (**B–E**) LSA-NRC was exposed to CatB, CatH, and CatS for 3 hours (**B**), 1 hour (**C**), or 15 min (**D)** followed by incubation with DR1 and DM for additional 3 hours, and (**E**) Mass spectrum of peptide eluted from DR1 when all the components of the cell-free system were simultaneously incubated together. (**F**) The background sample contains all of the cell-free system components except the LSA-NRC. The immunodominant peptide is labeled in red and LSA-NRC derived non-dominant epitopes are labeled in blue. Representative of four independent experiments/panel.

The next possibility that we examined was whether a difference in sensitivity to cathepsins might have given the HA epitope an advantage over the LSA-derived epitope. We pre-incubated LSA-NRC protein with our cathepsin mixture, CatB, CatH and CatS, for different lengths of time (3 h, 1 h, or 15 min), before adding or concurrently with DR1 and DM for epitope binding and selection. The outcome, as analyzed by MS, indicated that the LSA-derived dominant epitope was highly sensitive to the cathepsins (Fig. 4B–E). The dominant epitope could not be detected by mass spectrometry at all time points tested, even when all the components of the cell free system were concurrently incubated together (Fig. 4E). We could only detect LSA derived dominant epitope (in red) if the LSA-NRC protein was incubated with DR1 and DM first, and then exposed to the cathepsin mix (Fig. 4A). Similar control experiments were performed on H5N1-rHA1 protein supplementary Fig. 1 and ref. 33 indicating a slight remaining peak of the H5N1-HA1(259–274) peptide was detected by mass spectrometry even after three hours of pre-incubation with cathepsins in the absence of DR1. Thus, a difference in sensitivity to the cathepsins might have given the dominant epitope of HA1 protein a winning edge in gaining dominance. This difference is not due to differences in susceptibility of the dominant epitopes to the cathepsin digestion^33^, but perhaps the location of the epitopes, which is the C-terminus of the protein for LSA-NRC epitope, but a less accessible folded HA1 head for H5N1-HA1(259–274) epitope. Therefore, structural constraints might also contribute to the selection of dominant epitopes.

**Figure 5:**
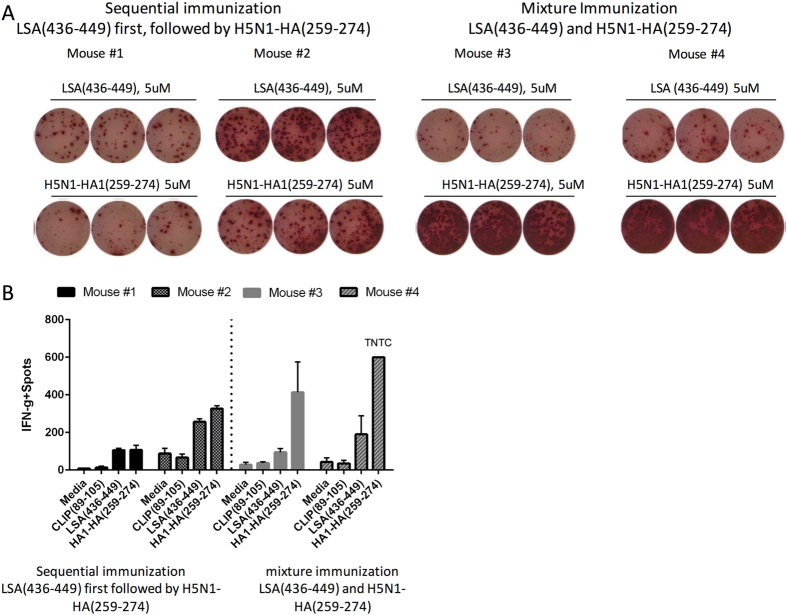
CD4 T cell responses to both H5N1-rHA1 and LSA-NRC when DR1 mice are sequentially immunized with the LSA-NRC dominant epitope first, followed by the H5N1-HA1 dominant epitope. (**A**–**B**) ELISPOT assay measuring IFN-γ production of T cells isolated from DR1-transgenic mice immunized first with LSA(436–449) and then immunized with HA(259–274) 2 days later (mouse #1 and #2). Or, IFN-γ production was measured by ELISPOT assay when mice were immunized with a mixture of LSA(436–449) and HA(259–274) in CFA (mice 3 and 4). Cells were stimulated with peptides for 24 h. (**B**) Error bars are defined as s.d. Representative of two independent experiments/panel. When the spots exceeded 600 the analyzer gave the error message TNTC (too numerous to count). Thus, those plates were considered to have over IFN-γ 600 spots.

The poor responsiveness to LSA-NRC-derived epitopes when co-immunized with H5N1-HA1 could not be attributed to lack of specific T cells because mice respond well to the LSA-NRC protein when injected alone Fig. 2D and ref. 21. To further examine if the LSA epitope might lose the competition to the H5N1-HA epitope in binding to the MHC II groove when both epitopes are simultaneously present, we immunized mice with the dominant epitopes from either protein sequentially, giving LSA-NRC a 2-days lead over rHA1, or mixed the two peptides to serve as controls. IFN-γ production by T cells isolated from immunized DR1-transgenic mice, were measured by ELISPOT assay (Fig. 5). If mice were first immunized with LSA(436–449), and two days later with HA(259–274), T cells responded to both antigens equally well (Fig. 5A, left panel). When mice were immunized with a mixture of the two peptides, the recall response to H5N1-HA(259–274) was prominent over the LSA(436–449) epitope (Fig. 5A, right panel). The summary of the data is shown in Fig. 5B. These findings show that the epitope hierarchy is likely established during competition for binding to the MHC II groove, and as such, any factor that can add to the longevity of an epitope or its more rapid capture might contribute to its emergence as dominant.

### Artificial spacer sequences designed to facilitate protein purification can be captured as the immunodominant epitope from recombinant proteins

As discussed above, we have previously determined the immunodominant epitope of a recombinant liver stage antigen of malaria falciparum (LSA-NRC). The dominant epitope of LSA-NRC, LSA(434–453) was located at the C-terminus of the protein (residues 434–443) and contained part of a spacer sequence artificially added to the protein for purification purposes (residues 444–453)^43^ (Supplementary Table 1). While the LSA sequence ends at Leucine 443, the immunodominant epitope includes the spacer sequence GGSGSP and four histidine residues. Importantly, the artificial epitope was the selected determinant *in vivo*, as verified by its ability to induce T cell responses in DR1 transgenic mice immunized with LSA-NRC in CFA (Fig. 2D), and in human volunteers immunized with LSA-NRC vaccine preparations^21^. A synthetic peptide that did not contain the spacer sequence, LSA(429–443) (VDELSEDITKYFMKL), did not stimulate T cells to proliferate or produce cytokines in the immunized subjects or mice. The peptide containing the spacer sequence, LSA(436–449) was the only peptide to recall a strong dose dependent proliferation.

**Figure 6:**
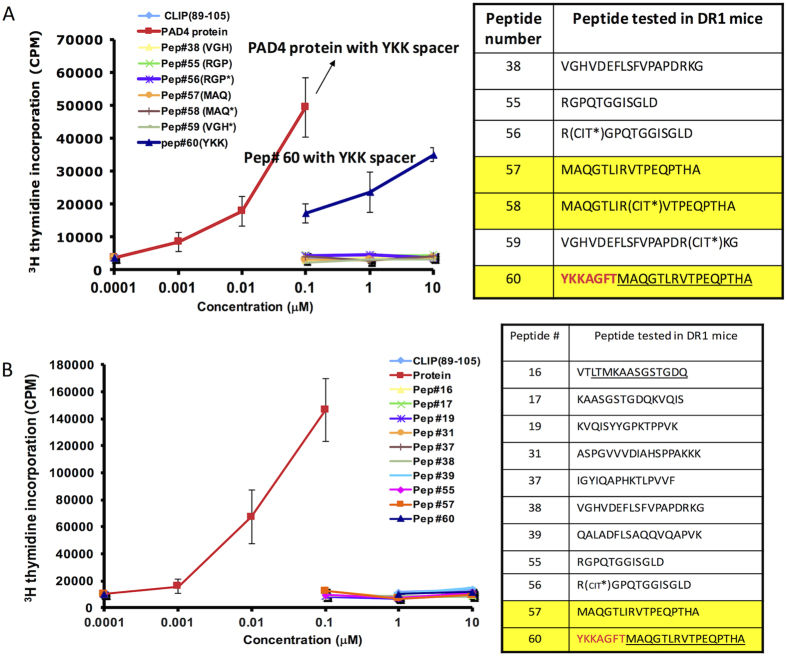
The spacer sequence inserted into the recombinant protein captured as the immunodominant epitope. (**A**,**B**) Proliferation of T cells isolated from DR1-transgenic mice immunized with recombinant PAD4-A protein in CFA at the base of tail. On day 8, cells were harvested from draining LNs and stimulated with proteins and peptides *in vitro*. (**A**) DR1 mice were immunized with recombinant PAD4-A protein, or **(B)** recombinant PAD4-B protein containing a different spacer sequence (shown in red). Cells were stimulated *in vitro* with either PAD4 proteins, CLIP(89–105), or peptides identified from PAD4-A, peptides Pep#16, #17, Pep#19, Pep#31, Pept#37, Pep#38, Pep #39, Pep#55 and its citrulinated (cit*) variant #56, Pep#57, peptide 59 (cit*), and YKK (Pep#60) *in vitro*. Cellular proliferation was measure by [3 H] thymidine incorporation. (CIT*) indicates citrullination of arginine in the synthetic peptides. The sequence of the native protein adjacent to the spacer sequence is underlined, and the spacer sequence is shown in red. Error bars are defined as s.d. Representative of five independent experiments/panel.

Another example of a new immunodominant determinant generated by adding a spacer sequence to a recombinant protein came from studying recombinant peptidylargine deimnase 4 (PAD4) protein. PAD4 is a Ca^2+^ dependent enzyme that catalyzes conversion of arginine residues to citrulline^46^. Autoantibodies to proteins containing citrulline residues as well as to the PAD4 enzyme itself are associated with human rheumatoid arthritis (RA)^47^. HLA-DR1 (HLA-DRB1*0101) and HLA-DR4 (HLA-DRB1*0401) belong to a family of specific HLA-DR1 alleles, referred to as the shared epitope alleles, that have a conserved amino acid motif present in the peptide binding groove and are associated with the development of RA^48^,^49^. To determine the PAD4 derived dominant epitopes, we used our cell free reductionist antigen processing system. Recombinant PAD4 protein was incubated with DR1 and DM followed by addition of CatB, CatH, and CatS. Peptides were eluted from DR1 and were analyzed by mass spectrometry. We identified eight peptides of PAD4 shown in Table 2. Immunogenicity of those epitopes were verified by harvesting T cells from the draining lymph nodes of DR1 transgenic mice immunized with PAD4 in CFA. Cells were challenged with all of the identified peptides *in vitro* separately. Tested peptides included synthetic peptides with and without modification of citrullinated arginine (marked as R(cit)*). Only peptide #60, among all tested, recalled a response (Fig. 6A). Strikingly, peptide #60 contained a portion of the N-terminal spacer sequence from the vector (YKKAGFT) and adjacent N-terminal PAD4 residues (MAQGTLRVTPEQPTHA). Notably, the spacer sequence added to this PAD4 protein is different from that added to LSA-NRC, (MSYYHHHHHHLESTSLYKKAGFT), and has been placed at its N-terminal end (Supplementary Table 1**, recombinant LSA-NRC**). The cell free system also identified a peptide comprised of the natural N-terminal sequence of PAD4 alone, peptides #57 (MAQGTLRVTPEQPTHA). We tested peptides #57 and its citrullinated version, peptides #58 (MAQGTLR(cit)*VTPEQPTHA). Neither peptide stimulated T cells from PAD4 protein immunized mice to proliferate or secrete cytokines upon challenge. This shows that by addition of the YKKAGFT spacer sequence, the immunogenicity of the PAD4-A protein has shifted from it natural sequences to an artificial epitope that contains the spacer sequence.

To ascertain reproducibility of this phenomenon, another recombinant PAD4 protein (PAD4-B), which was identical to PAD4-A except that it contained a different spacer sequence MGSSHHHHHH**GSAEGSS** (Supplementary Table 2**, recombinant PAD4-B**) was used for immunizing DR1 transgenic mice. When mice were immunized with PAD4-B, and challenged *in vitro* with PAD4-B protein or all the individual peptides shown in the figure, T cells responded to the PAD4-B protein only, but not to peptide #60 (Fig. 6B). These results emphasize how small changes in protein sequence and/or structure can disturb the natural processing and immunodominant epitope selection. As such, design of recombinant protein immunogens requires careful evaluation before any attempt at their use in clinical trials.

## Discussion

We investigated epitope hierarchy by using our reductionist antigen processing system first, and then confirming the findings *in vivo* and made two astonishing observations. First, that immunodominance of an epitope is not absolute: the immunodominant epitope of one antigen might become subdominant or cryptic when other more robust epitopes are simultaneously present. Second, three recombinant proteins, LSA-NRC, and two different constructs of PAD4, having three distinct spacer sequences for attachment of a His-tag to the C- or N-termini of antigens, displayed an unpredictable characteristic in epitope immunodominance by distorting the physiological epitope selection processes. In all three recombinant proteins, new epitopes that included the spacer sequences emerged as immunodominant!

Importantly, in case of LSA-NRC, the protein was used as a vaccine candidate in humans, and it appeared that vaccinated individuals selectively recognized the spacer containing epitope as the dominant epitope^21^. With PAD4 proteins, the study was done in mice indicating that for both proteins, the spacer tags were recognized as the immunodominant epitopes. These findings are of great significance and should alarm researchers that small changes in proteins used as immunogens can have undesirable effects. Undoubtedly, many vaccine efforts must have been failed simply by not realizing that the T cell responses generated by vaccination were targeted to new epitopes generated by addition of spacer sequences, rather than the physiological epitopes of the natural pathogens. Thus, epitope hierarchy plays a pivotal role in designing new and combination vaccines. In light of the notion that the spacer sequences were identified by our cell free reductionist antigen processing system, despite its contrived nature, and by the vaccinated volunteers^21^, we wish to emphasize the power of this system and hope that it can be widely used for testing antigens designed for vaccine trials.

We find that epitope hierarchy can occur among epitopes of the same protein, or immunodominant epitopes of different proteins. It appears that while proteins are being simultaneously processed, a race for binding to the groove of MHC II is ongoing. As expected, a combination of factors can contribute to the emergence of one epitope over another as dominant. For example, loss of dominance of the LSA-derived epitope to the H5N1-HA1-derived dominant epitope during processing might be explained by the distinct locations of each epitope within the protein structure, as the dominant epitopes of each protein have major differences in accessibility. LSA epitope is located at the C-terminus of the protein and therefore, more likely to be readily accessible to be captured by MHC II and to proteolysis. The opposite is true about H5N1-HA1-derived dominant epitope, which is centrally located and is rather protected. Sensitivity to digestion by cathepsins seems to play a critical role for LSA-NRC-derived epitope as the epitope becomes undetectable by mass spectrometry when briefly exposed to the mixture of cathepsins prior to incubation with DR1 and DM in our cell free system. For this epitope to be successfully captured by DR1, we must let DR1 to capture it first, before exposure to the cathepsins mixture. A slightly different case is observed for H5N1-HA1 protein: traces of the dominant epitope of H5N1-HA1 protein remains while the protein is exposed to the cathepsin mixture in the absence of DR1 and DM^33^. Hence, it is likely that H5N1-HA1(259–274) epitope wins the competition in the race for binding to the MHC II groove, because it is less accessible to digestion by the cathepsins present within antigen processing compartments. The sensitivity of the epitope itself to digestion by cathepsins is no different than that of LSA(434–453)^33^. As synthetic peptides, both epitopes are equally sensitive to destruction by cathepsins, but when embedded in the protein structure, they display slight differences in sensitivity to cathepsins.

The concept of epitope accessibility also sets the rules for the emergence of epitopes that include the spacer sequences as dominant epitopes. Accessibility of an epitope combined with the right amino acid sequences to form DM resistant complexes with MHC class II, can pose dominance over the other epitopes by being presented at higher quantities to T cells^33^. The above findings highlight the underlying principles for immunodominance for MHC class II by providing clear evidence for the contribution of factors intrinsic to antigenic structure or epitope accessibility. Notwithstanding, these observations suggest that determinant *hierarchy* is established in the antigen processing compartments.

Our findings challenge the current dogma that accepts, ‘*once immunodominant, always immunodominant’*^50^. It is believed that an immunodominant epitope would emerge as dominant no matter where it might be placed within a carrier protein^12^. Factors such as kinetic stability, or DM resistance^51^ are important contributors to immunodominance, although low affinity peptides have also been reported as dominant^52^. Thus, our new finding present a cautionary tale to vaccine designers^35^,^53^, warning against the assumption that placement of dominant determinants in any context would not affect their selection as dominant epitopes, or mixing several vaccines together might result in effective immunity against all the immunogens in the mix.

## Methods

### Production of Recombinant Proteins

Soluble HLA-DR1*0101 was produced as described previously in baculovirus-transduced insect cells^54^. Soluble HLA-DM was expressed in the same manner and affinity-purified with M2 mAb Sepharose (Sigma) at pH 6.0 through the FLAG tag placed on the α chain C-terminus. H5N1-rHA1 from strain A/Vietnam/1203/2004 was purified from 293 cells (eEnzyme). The expression, purification and biochemical and immunological characterization of *E. coli*–produced, GMP-manufactured LSA-NRC antigen has been described previously^43^. Recombinant N-terminal 6xHis tagged PAD4 (NP_036519) in the Gateway pDEST17 vector (Thermo Fisher) was expressed and purified from BL21(DE3) pLysS competent cells as previously described^34^. Recombinant PAD4-B with MSYYHHHHHH**LESTSLYKKAGFT** His and spacer sequence was expressed in *E. coli* (Cayman Chemical).

#### Peptides synthesis

Peptides HA(259–274) (SNGNFIAPEYAYKIVK) and PAD4 peptides were synthesized by Elim Biopharmaceuticals to >90% purity. All other peptides were synthesized by Peptide2.0, at 90–95% purity, as determined by reverse-phase HPLC, and were confirmed by mass spectrometry.

#### Proliferation and cytokine assays

HLA-DR1 (DRB1*0101) transgenic mice (originally obtained from Dr. Denis Zaller, Merck) expressing a fusion product of the DR1 binding groove and the membrane proximal domain of I-E molecules^55^ were backcrossed to MHC class II KO mice for 12–16 generations to eliminate endogenous class II proteins (I-A^f^) and then were inbred to homozygosity. DR1 transgenic mice were immunized with 50 μg proteins (LSA-NRC, H5N1-rHA1, or PAD4-A or PAD4-B) or 50 nmoles peptides in Complete Freund’s Adjuvant (CFA) at the base of tail. After 8–10 days, cells were harvested from the draining lymph nodes and incubated with peptides and protein in multiple concentrations for 3 days in culture before adding [^3^H] thymidine (Amersham plc). Cells were then harvested and counted after a further 18–20 h culture. For IFN-γ ELISPOT assay (BD Biosciences), cells (8 × 10^5^) from the draining lymph nodes were incubated with peptides (5 μM), or proteins (0.25 μM) for 24 h or 48 h. After the incubation, ELISPOT plate was analyzed with the CTL Immunospot Analyzer plate reader and software (Cellular Technology Limited). Of note is that the analyzer could not read the spots when their numbers exceeded 600 and gave the error message TNTC (too numerous to count). Thus, those plates were considered to have over IFN-γ 600 spots.

*Peptide dissociation assays*: were performed as described previously^45^. A stock solution of DR1 (10 μM) was incubated with fluorescence-labeled peptide (150 μM) in PBS/NaN_3_ for 3 days at 37 °C to yield maximal loading. Unbound fluorescent peptide was removed by spin columns at pH 5 citrate phosphate/NaN_3_. Dissociation of fluorescent peptide/DR complexes (1 μM) was monitored in samples incubated for different length of times in the presence of excess unlabeled competitor peptide (100 μM) with or without DM (1 μM). Unbound peptide was removed by spin columns (Sephadex G-50 equilibrated with PBS/0.1% filtered non-fat milk) at pH 7.4. Fluorescence emission of the FITC–peptide–DR complexes was measured at 25 °C and 514–516 nm with excitation at 492 nm on a Fluoromax3 spectrofluorometer (Horiba Jobin-Yvon, Kyoto, Japan) with a slit width of 2 nm.

### Mass Spectrometry

#### Sample preparations for MS analyses

HLA-DR1, protein antigens, and HLA-DM were incubated in citrate phosphate buffer (pH 5.0) at 37 °C for 3 h before adding Cat B (bovine spleen, Sigma), Cat H (human liver, Calbiochem), and Cat S (human recombinant produced in *E. coli*, Calbiochem) in 6 mM L-Cysteine and 4 mM EDTA for an additional 2 h. After digestion, the pH was adjusted to 7.4, 10 μM iodoacetamide was added, and DR1 was immunoprecipitated with HLA-DR specific mAb (L243) conjugated Sepharose beads. For protein pre-digestion assay, proteins were incubated in citrate phosphate buffer (pH 5.0) at 37 °C for 15 min, 1 h, or 3 h with cathepsins B, H, and S and 6mM L-cysteine and 4 mM EDTA. Then, DR1 and DM was added and incubated for additional 3 h. After the incubation, DR1 binding peptides were prepared as described above.

#### vMALDI-LTQ analysis

Peptides were desalted using zip-tip C18 (Millipore), resuspended in 5 μL of 50% ethanol and 0.1%TFA and 0.5 μl spotting onto a 96 well stainless vMALDI plate. Peptide spots were dried, covered with 0.5 μL 40–50 mg/ml 2,5-Dihydroxybenzoic acid (DHB) in 50% ethanol and 0.1%TFA and analyzed on the vMALDI-LTQ ion trap mass spectrometer (Thermo Scientific) with 400 micron optic fiber, using Tune Plus 2.2 Xcalibur 2.0 SR2 vMALDI LTQ 2.2 with Microsoft Windows XP service pack 2 software. Spectra were manually acquired using CPS plate motion, 2 microscans and 0 sweep scans with automatic scan filtering (ASF) feature set at 3000 for survey scans (MS) and 200 for CID fragmentation scans (MS2 and MS3). Survey scans (10–15 MS spectra) were accumulated on a 1100–4000 m/z mass range to find peptide masses of interest (i.e, unique peptides from the recombinant protein containing samples). These target peptides were fragmented by collision induced dissociation (CID) and 15–25 fragmentation spectra (MS2) were accumulated for sequence analysis. Several fragment ions were also subjected to CID fragmentation producing MS3 spectra to confirm peptide sequences. Monoisotopic masses from accumulated MS2 and MS3 spectra were extracted and searched using Sequest algorithm in Bioworks 3.3.1 SP1 (Thermo Scientific) against custom-built database, containing all proteins presented in our cell free antigen processing system, including additional recombinant proteins and other common potentially contaminant proteins (e.g. keratins, albumin, etc.). Search criteria consisted of: no enzyme specified; mass tolerance of 1.2 Da and 1.0 Da for peptide and fragment ions; respectively; annotating ions a, b and y ions; cysteine carboamidomethylated, mono- and di-oxidized methionine as variable modifications; and 64 Da neutral loss for methionine. Peptide identifications were filtered at a XCorr >1.5 for singly charged ions.

## Additional Information

**How to cite this article:** Kim, A.R. *et al*. Distorted Immunodominance by Linker Sequences or other Epitopes from a Second Protein Antigen During Antigen-Processing. *Sci. Rep.* **7**, 46418; doi: 10.1038/srep46418 (2017).

**Publisher's note:** Springer Nature remains neutral with regard to jurisdictional claims in published maps and institutional affiliations.

## Supplementary Material

Supplementary Material

## Figures and Tables

**Table 1:** Epitopes from H5N1-HA1 (aa-1–345) restricted to DR1.

MEKIVLLFAIVSLVKSDQICIGYHANNSTEQVDTIMEKNVTVTHAQDILEKKHNGKLCDLDGVKPLILRDCSVAGWLLGNPMCDEFINVPEWSYIVEKANPVNDLCYPGDFNDYEELKHLLSRINHFEKIQIIPKSSWSSHEASLGVSSACPYQGKSSFFRNVVWLIKKNSTYPTIKRSYNNTNQEDLLVLWGIHHPNDAAEQTKLYQNPTTYISVGTSTLNQRLVPRIATRSKVNGQSGRMEFFWTILKPNDAINFE**SNGNFIAPEYAYKIVK**KGDSTIMKSELEYGNCNTKCQTPMGAINSSMPFHNIHPLTIGECPKYVKSNRLVLATGLRNSPQRERRRKKHHHHHH
SNGNFIAPEYAYKIVK:DR1 binding immunodominant epitope.

**Table 2:**
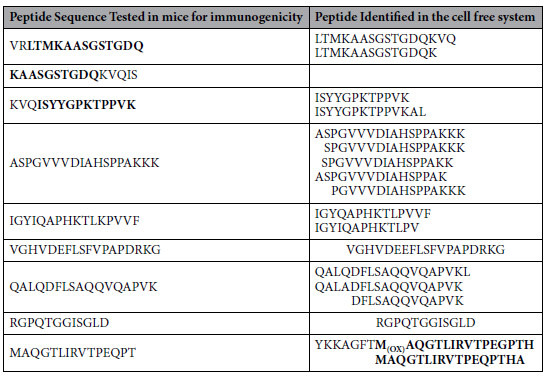
The summary of PAD4 derived epitopes identified using the reductionist cell-free antigen processing system. (ox) indicates the oxidation of Methionine.
