# Supplementary material for: Distorted Immunodominance by Linker Sequences or other Epitopes from a Second Protein Antigen During Antigen-Processing
Source: Sci Rep. 2017 Apr 19;7:46418. doi: 10.1038/srep46418 (PMC5396073; doi:10.1038/srep46418)
Supplement: Supplementary Material [file srep46418-s1.pdf]

Legends to Supplementary Tables and Figure for the manuscript, SREP-16-42913A,

Title:

Distorted Immunodominance by Linker Sequences or other Epitopes from a Second Protein Antigen During Antigen-Processing.

Authors

AeRyon Kim, Tatiana N. Boronina, Robert N. Cole, Erika Darrah, and Scheherazade Sadegh-Nasseri

Legends to Supplementary Tables and Figure

Supplementary Table I. LSA-NRC derived peptides identified by the cell-free system are shown in bold. Peptides in blue and red were identified when LSA-NRC was pre-incubated with DR1 and DM first and then digested with cathepsins. The three peptides used to recall responses in vitro included the peptide with the spacer sequence (GGSGSP), $LSA_{436\text{-}449}$, in addition a peptide was identified by the cell free system shown in blue $LSA_{323\text{-}337}$), and another $LSA_{429\text{-}443}$ that spanned the C-terminus of the native LSA protein but not including the spacer sequence.

Supplementary table 2. Amino acid sequences of two recombinant PAD4 proteins with two different spacer sequences.

Peptide labeled in red indicates the linker sequences inserted between His tag sequence and amino acid sequence of PAD4. PAD4 derived epitopes identified from the cell-free system are labeled in blue.

Supplementary Figure 1. The repertoire of H5N1-rHA1-derived peptides eluted from HLA-DR1 changes when H5N1-rHA1 is treated with Cathepsins B, H, and S prior to incubation with HLA-DR1 and DM.

A-C. The mass spectrum of peptides eluted from HLA-DR1 (enlarged spectrum between m/z 1100-2900 Da) when denatured H5N1-rHA1 (A/Vietnam/1203/2004) was first incubated with HLA-DR1 and HLA-DM and then exposed to cathepsins B, H, and S (**A)**, or when denatured H5N1-rHA1 is first exposed to cathepsins B, H, and S for 3h (**B)** and then incubated with HLA-DR1 and DM. The background spectrum is shown in (**C**). Mass species highlighted in red represent H5N1-rHA1 fragments containing the HLA-DR1 restricted immunodominant $HA_{259\text{-}274}$ epitope.

LSA-NRC amino acid sequence:

MGTNSEKDEIIKSNLRSGSSNSRNRINEEKHEKKHVLSHNSYEKTKNNENNKFFDKDKELTMSNVK
NVSTNFKSLLRNLGVSENIFLKENKLNKEGKLIEHIINDDDDKKKYIKGQDENRQEDLEEKAAEQQSD
LEQERLAKEKLQERLAKEKLQEQQRDLEQRKADTKKNLERKKEHGDVLAEDLYGRLEIPAIELPSENE
RGYYIPHQSSLPQDNRGNSRDSKEISIIEKTNRESITTNVEGRRDIHKGHLEEKKDGSIKPEQKEDKSA
DIQNHTLETVNISDVNDFQISKYEDEISAEYDDSLIDEEEDDEDLDEFKPIVQ**YDNFQDEENIGIYK**EL
EDLIEKNENLDDLDEGIEKSSEELSEEKIKKGKKYEKTKDNNFKPNDKSLYDEHIKKYKNDKQVNKEKE
KFIKSLFHIFDGDNEILQIVDELS**EDITKYFMKLGGSGSPHHHH**HH

	Mass $_{monoisotopic}$	Sequence
Peptides identified using the cell-free system	1747	YDNFQDEENIGIYK
	1877	YDNFQDEENIGIYKE
	1821	EEDDEDLDEFKPIVQ
	1951	EEEDDEDLDEFKPIVQ
	2278	EDITKYFMKLGGSGSPHHHH
	2295	EDITKYFM$_{OX}$KLGGSGSPHHHH

Peptides used for proliferation and cytokine assay	Sequence
$LSA_{323-337}$	VQYDNFQDEENIGIY
$LSA_{429-443}$	VDELSEDITKYFMKL
$LSA_{436-449}$	ITKYFMKLGGSGSP

Peptide modification: M_{ox} (oxidation of methionine)

Supplementary Table I. Recombinant LSA-NRC amino acid sequence

Recombinant PAD4 #1:

MSYYHHHHHH**LESTSLYKKAGFTMAQGTLIRVTPEQPTHA**VCVLGTLTQLDICSSAPEDCTSFS**INASPGVVVDIAHSPPAKKK**S
TGSSTWPLDPGVEVT**LTMKAASGSTGDQKVQISYYGPKTPPVK**ALLYLTAVEISLCADITRTGKVKPTRAVKDQRTWTWGPCG
QGAILLVNCDRDNLESSAMDCEDDEVLDSEDLQDMSLMTLSTKTPKDFFTNHTLVLHVARSEMDKVRVFQATRGKLSSKCSVVL
GPKWPSHYLMVPGGKHNMDFYVEALAFPDTDFPGLITLTISLLDTSNLELPEAVVFQDSVFRVAPWIMTPNTQPPQEVYACSIFE
NEDFLKSVTTLAMKAKCKLTICPEEENMDDQWMQDEME**IGYIQAPHKTLPVVF**DSPRNRGLKEFPIKRVMGPDFGYVT**RGPQ**
TGGISGLDSFGNLEVSPPVTVRGKEYPLGRILFGDSCYPSNDSRQMH**QALQDFLSAQQVQAPVK**LYSDWLS**VGHVDEFLSFVP**
APDRKGFRLLLASPRSCYKLFQEQQNEGHGEALLFEGIKKKQQKIKNILSNKTLREHNSFVERCIDWNRELLKRELGLAESDIIDIP
QLFKLKEFSKAEAFFPNMVNMLVLGKHLGIPKPFGPVINGRCCLEEKVCSLLEPLGLQCTFINDFFTYHIRHGEVHCGTNVRRKPF
SFKWWNMVP

Recombinant PAD4 #2:

MGSSHHHHHH**GSAEGSS**AQGTLIRVTPEQPTHAVCVLGTLTQLDICSSAPEDCTSFSINASPGVVVDIAHSPPAKKKSTGSSTWP
LDPGVEVTLTMKAASGSTGDQKVQISYYGPKTPPVKALLYLTAVEISLCADITRTGKVKPTRAVKDQRTWTWGPCGQGAILLVNC
DRDNLESSAMDCEDDEVLDSEDLQDMSLMTLSTKTPKDFFTNHTLVLHVARSEMDKVRVFQATRGKLSSKCSVVLGPKWPSHY
LMVPGGKHNMDFYVEALAFPDTDFPGLITLTISLLDTSNLELPEAVVFQDSVFRVAPWIMTPNTQPPQEVYACSIFENEDFLKSVT
TLAMKAKCKLTICPEEENMDDQWMQDEMEIGYIQAPHKTLPVVFDSPRNRGLKEFPIKRVMGPDFGYVTRGPQTGGISGLDSF
GNLEVSPPVTVRGKEYPLGRILFGDSCYPSNDSRQMHQALQDFLSAQQVQAPVKLYSDWLSVGHVDEFLSFVPAPDRKGFRLLL
ASPRSCYKLFQEQQNEGHGEALLFEGIKKKQQKIKNILSNKTLREHNSFVERCIDWNRELLKRELGLAESDIIDIPQLFKLKEFSKA
EAFFPNMVNMLVLGKHLGIPKPFGPVINGRCCLEEKVCSLLEPLGLQCTFINDFFTYHIRHGEVHCGTNVRRKPFSFKWWNMVP

Supplementary Table II. Sequence of the two different PAD4 used in this study. Epitopes identified by the cell free processing system are highlighted in blue, with the spacer sequences in red.

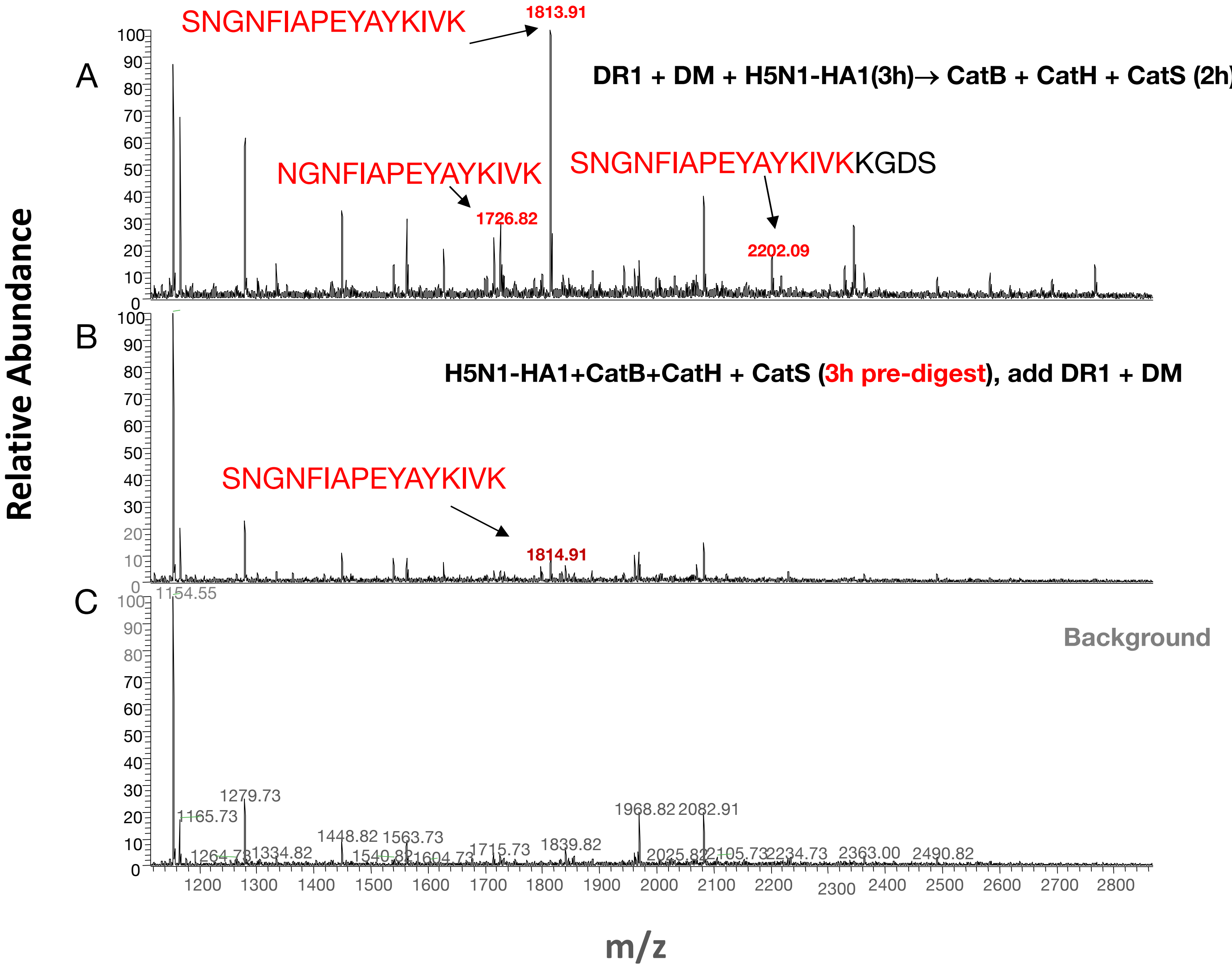

Supplementary Figure 1
